# Scanning Electron Microscopic Analysis of Deformation of TruNatomy File Systems: An Ex-vivo Study

**DOI:** 10.7759/cureus.44183

**Published:** 2023-08-27

**Authors:** Harish Selvaraj, Jogikalmat Krithikadatta, Kavalipurapu Venkata Teja

**Affiliations:** 1 Conservative Dentistry and Endodontics, Saveetha Dental College and Hospitals, Saveetha Institute of Medical and Technical Sciences, Saveetha University, Chennai, IND; 2 Department of Conservative Dentistry and Endodontics, Mamata Institute of Dental Sciences, Hyderabad, IND

**Keywords:** trunatomy, smear layer, root canal, ni-ti instruments, hero shaper

## Abstract

Aim

The aim of the study was to assess the instrument deformation following the usage of the TruNatomy (Dentsply Sirona, USA) file system in extracted premolars using Scanning Electron Microscopy (SEM).

Materials and Methods

For the present study, 84 extracted mandibular bicuspids were selected. The teeth were divided into two groups: Group 1, TruNatomy (n=42), and Group 2, Hero Shaper (Micro Mega, France) (n=42). The samples were shaped with 0.03 taper to size 36 with TruNatomy instruments and 0.04 taper to size 30 with Hero Shaper instruments. 5 ml of 5.25% Sodium Hypochlorite (NaOCl) and 2 ml of 17% ethylenediaminetetraacetic acid (EDTA) were used for the final irrigation of root canals. The master apical file was used to instrument seven teeth samples for assessing the safety of the instruments after multiple uses and autoclaving. So, a total of six files per group was used for the analysis of any distortions, cracks or micro-fractures after instrumentation of 42 teeth, at the tip (D0) and 5 mm from the tip (D5) of the rotary file under SEM at 500x magnification.

Statistical analysis

To determine the significance between the groups, the Mann-Whitney U-test was applied.

Results

The mean surface wear of the instruments, at the tip (D0) in Group 1 was 1.2857 and in Group 2 was 1.4762. The mean spiral distortion of the instruments, at the tip (D0) in Group 1 was 1.1905 and in Group 2 was 1.4286. A statistically significant difference (P<0.05) was observed between the groups for surface wear and spiral distortion at the tip of the file (D0). There was no significant difference between groups for surface wear and spiral distortion values of the instruments at 5 mm from the tip (D5) (P>0.05).

Conclusion

The instrument distortion of the rotary file systems analysed was minimal following the biomechanical preparation of seven mandibular bicuspids without root curvature, using a single file. Therefore, both rotary file systems can be considered safe.

## Introduction

Biomechanical preparation provided direct access for an irrigant to effectively cleanse and disinfect the walls of the root canals [1]. Various instrumentation systems have been available for years for preparing the root canal [2]. The trend in shaping was inclined toward the usage of rotary nickel-titanium (NiTi) systems, which enhanced the treatment outcomes [3]. A wide range of instrumentation system types, designs, and surface modifications have been done to instruments to prevent the inherent cyclic and torsional fatigue [4]. NiTi alloys with various surface treatments have been produced in recent years with improved mechanical properties and clinical efficiency [5]. Thermal processing methods changed NiTi alloy transition temperatures [6]. The inherent properties of nickel-titanium endodontic instruments have been found to be enhanced by heat treatment by altering their transformation behaviour [7]. The preparation techniques have also been modified to prevent inherent procedural errors [8]. Currently, a wider range of instruments with different designs, cross-sections, and transformations of the original alloy are available [9]. In recent years, there have been lots of improvements in engine-driven rotary instrumentation systems [10]. A recent systematic review investigated the NiTi instruments and identified several factors that could influence the stress generated within the rotary instrumentation system [11]. Recently, the kinematics of motion has changed drastically with the rotary NiTi instrumentation systems. Various movement kinetics, such as rotation, reciprocation, and adaptive instrumentation systems have evolved to improve the performance and safety during instrumentation in the root canals [12].

Nickel-titanium rotary instruments tend to separate frequently [13]. The irregularities present on the surface of the instrument tended to cause separation. The rotary instruments are subjected to various compression and tension forces in the arched portion of root canals, leading to instrument separation [14]. Instrument separation frequently occurred when there were more signs of instrument distortion [15]. Inherent cracks and the risk of separation were higher when a distorted instrument rotated inside the canal [16]. Separation was more frequent with NiTi file systems compared to stainless steel hand files, with a range of 0-23% [14]. Multiple usages of distorted file systems increased the incidence of separations [15]. So, for a practicing clinician, it was important to know the clinical instrument-related factors for separation. Hence, the current study aimed to evaluate the instrument distortion of TruNatomy (Dentsply Sirona, USA) file systems in mandibular bicuspids after multiple uses.

## Materials and methods

The study had received prior clearance from the Saveetha University Institutional Human Ethical Committee (IHEC/SDC/ENDO/-2005/21/280). The sample size was calculated based on Paqué et al. [17], using G Power software version 3.1 (1-β = 90%, α = 0.05). Eventually, a sample size of 84 was achieved. Eighty-four freshly removed human mandibular bicuspid teeth were obtained for the current study. Teeth with complete formation of the root, curvatures less than 10 degrees, intact which underwent extraction for orthodontic or periodontal reasons had been selected. Teeth with immature apices and calcifications were excluded. Soft tissue attached to the tooth surfaces was removed using a curette. Teeth specimens were evaluated for the possibility of extra canals by using angulated radiographs.

The access cavity for endodontic therapy was initiated using a small-sized round bur BR-31 (Mani Inc, Japan) with an air-rotor handpiece under water coolant. The canals were worked to the apex using a #10 K-file (Mani Inc, Japan) until the file tip was visible through the apex. The teeth were then randomly divided into two groups: Group 1 (Dentsply TruNatomy Files) (n=42) and Group 2 (Hero Shaper Files) (n=42). Glide path files were used for Group 1, while manual K-files were used for Group 2 for glide path preparation. Root canal preparation was performed using a low-speed endodontic handpiece (Dentsply X-Smart Plus, Dentsply Sirona, USA), following the manufacturer's recommendations for torque and speed settings. Root canals were intermittently irrigated using a 30 gauge side-vented needle and 5.25% sodium hypochlorite (NaOCl) (Parcan, India). For each new file used, irrigation was carried out using 2 ml of 5.25% NaOCl and 2 ml of 17% ethylene diamine tetraacetic acid (EDTA) solution (Endo Solution, Cerkamed, India). Finally, the root canals were irrigated with 5 ml of 5.25% sodium hypochlorite, followed by rinsing with distilled water and drying with absorbent paper points (Meta Absorbent Paper Points, Meta Biomed, Korea). The samples were shaped with a 0.04 taper to size 30 with Hero Shaper instruments and a 0.03 taper to size 36 with TruNatomy instruments. After each sample instrumentation, the rotary files were autoclaved and cleaned with gauze soaked in 70% alcohol. All the instrumented files were examined under SEM (JSM-IT800, Jeol, USA). Double-sided carbon tape was used to mount the files onto 23 mm SEM specimen mount stubs. A line was drawn on the handle of the first side of the file with permanent ink, ensuring that the same region of the file was examined in multiple evaluations. The files were scanned at a magnification of 500x. They were examined for surface wear and spiral distortion at two locations: the tip (D0) and 5 mm from the tip (D5), using the scoring criteria established in a previous study [18]; (1) The long axis of the file has undistorted spirals, which means that there are no stretching or compressing and no wear on the surface being examined, (2) The long axis of the file has a distorted spiral, which means that there are one to three defective or worn areas on the surface being examined, (3) The long axis of the file has more than one distorted spiral, which means that there are four or five worn areas on the surface being examined, (4) The spiral has significant wear and the surface under examination has more than five worn regions [18].

Statistical analysis

Using SPSS Statistics for Windows, Version 23.0 (IBM Corp., Armonk, NY), the data were statistically analysed. The significance between the groups was determined using a Mann-Whitney U-test.

## Results

**Figure 1:**
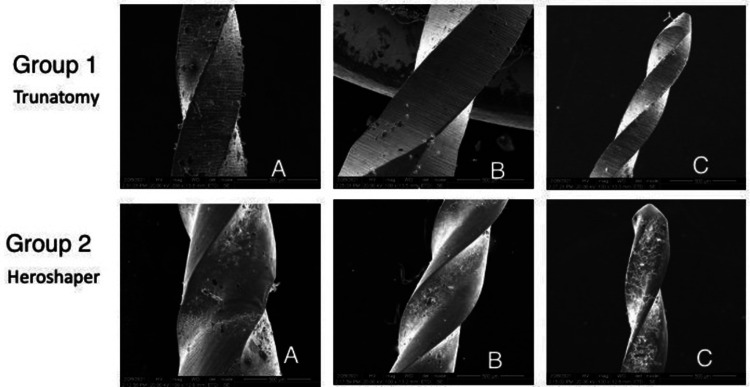
SEM images of TruNatomy and HeroShaper files following instrumentation The images show the presence of defects at 5 mm from the tip A & B and at the tip C (500X magnification). 1) Presence of defects at 5 mm from the tip of the instrument, Group 1: A & B and at the tip Group 1: C of TruNatomy file (36/03) used (groove and irregular edge). 2) Presence of defects at 5 mm from the tip of the instrument, Group 2: A & B and at the tip Group 2: C of Hero Shaper file (30/04) used (micro-cavity, groove and irregular edge).

The mean surface wear of the instruments at the tip (D0) was 1.2857 in Group 1 and 1.4762 in Group 2. The mean spiral distortion of the instruments at the tip (D0) was 1.1905 in Group 1 and 1.4286 in Group 2. A statistically significant difference (P<0.05) was observed between the groups for surface wear and spiral distortion at the tip of the file (D0). Group 2 exhibited higher surface wear and spiral distortion compared to Group 1 at the instrument tip (D0) (Table 1) (Figure 1). The mean surface wear of the instruments at 5 mm from the tip (D5) was 1.3333 in Group 1 and 1.5714 in Group 2. The mean spiral distortion of the instruments at 5 mm from the tip (D5) was 1.2857 in Group 1 and 1.5476 in Group 2. There was no significant difference between the groups for surface wear and spiral distortion values of the instruments at 5 mm from the tip (D5) (P>0.05) (Table 1) (Figure 1).

**Table 1:** Surface wear and spiral distortions of the files at tip (D0) and 5 mm (D5) Surface wear and spiral distortions of the files at tip (D0) and 5 mm (D5) of Groups 1 (TruNatomy) and 2 (Hero Shaper).

Parameters Assessed	Groups	N	Mean	Std. Deviation	P- Value
Surface Wear at Tip	Group 1	42	1.2857	.45723	.004
	Group 2	42	1.4762	.50549	
Surface Wear at 5mm	Group 1	42	1.3333	.47712	.096
	Group 2	42	1.5714	.50087	
Spiral Distortion at Tip	Group 1	42	1.1905	.39744	.000
	Group 2	42	1.4286	.50087	
Spiral Distortion at 5mm	Group 1	42	1.2857	.45723	.006
	Group 2	42	1.5476	.50376	

## Discussion

According to recent studies, there was no significant difference in the clinical fracture incidence between different instrumentation kinematics. The number of uses was found to be more critical in preventing instrument separation than the kinematics [18,19]. Therefore, the aim of the current study was to evaluate the instrument distortion during the instrumentation of premolars without root curvature. Significant differences were observed in the surface wear and spiral distortion of files at the tip (D0), while no differences were found at 5 mm from the tip (D5). The study results demonstrated less or no surface distortions with the TruNatomy file system compared to the Hero Shaper system. Literature evidence suggests that Hero Shaper can be used as a baseline for evaluating experimental instrument systems, and at .04 tapers, Hero Shaper file systems appeared to be safer [20]. The present study revealed micro-cracks with the Hero Shaper file system, whereas these distortions were less evident with the TruNatomy system. The observed micro-cracks with Hero Shaper files may be attributed to the narrow elasticity range resulting from the altered grain structure of these file systems [21]. The Hero Shaper instrument featured a variable helical angle of cutting edges from the tip to the shank, an adapted pitch, taper-dependent pitch variation, a positive rake angle, a large inner core, and three cutting edges. This design led to low flexibility and increased stress on the dentin during instrumentation, which would have resulted in the generation of more micro-cracks [22]. The TruNatomy files are designed with specific characteristics that contribute to their enhanced performance. These features include a special heat-treated 0.8 mm NiTi wire and an off-centred parallelogram cross-sectional design. The wire consists of a mixture of austenite, martensite, and R-phase at 22 degrees Celsius, providing its unique properties [23]. The slim NiTi wire, shorter pitch length, post-machining heat treatment used, and predominance of the martensite phase contribute to the increased resistance of the TruNatomy files to distortion. Additionally, TruNatomy files demonstrate the ability to undergo both plastic and elastic deformation prior to fracture, further enhancing their durability and performance [24].

Multiple usages of NiTi systems and have observed increased instrument separation in rotary NiTi files with inherent surface defects [25]. Autoclaving is a mandatory step for sterilising NiTi files to minimize the risk of cross-contamination. However, sterilisation through autoclaving can lead to an increase in surface roughness values as the number of autoclaving cycles increases. A previous study documented increased surface degradations and reduced cutting efficiency in autoclaved file systems [26]. These degradations were more severe when the instruments were exposed to NaOCl and EDTA liquids sequentially after autoclaving [27]. The surface roughness of the rotary file system increased after exposure to NaOCl and 17% EDTA, which could cause areas of stress concentration and crack formation, and reduce the fatigue resistance [28]. In the present study, each instrument was autoclaved before being used for another specimen, ensuring a clinically reliable study. The results demonstrated safe instrumentation even after seven uses.

The limitation of the study was that the assessments were carried out with single-rooted teeth. In clinical scenarios, extreme curvatures were more evident in multi-rooted teeth. Although the preparation taper and sizes were standardized in the current study, the diameter and metallurgy of the instruments used were different, which might have led to varied results. The influence of the access cavity designs and the types of inherent distortions caused were not assessed. Hence, future studies should concentrate on assessing molars with complicated anatomies and curvatures with a standardized protocol that simulates an actual clinical scenario.

## Conclusions

Based on the findings of this study comparing TruNatomy and Hero Shaper file systems for instrument distortion analysis, it was observed that TruNatomy exhibited fewer instrument distortions compared to Hero Shaper. Both systems were determined to be safe, indicating that neither TruNatomy nor Hero Shaper posed significant risks or complications during the instrumentation of the samples without root curvature and sterilization after each use, up to seven times. However, the reduced instrument distortions observed with TruNatomy suggest potential advantages in terms of overall procedural effectiveness and efficiency. Further research and clinical studies are warranted to validate these findings and ascertain if the observed benefits of TruNatomy in this specific context are consistent across different tooth types, root canal complexities, and clinical scenarios.
